# An amplicon-based sequencing approach for Usutu virus characterization

**DOI:** 10.1186/s12985-024-02426-7

**Published:** 2024-07-23

**Authors:** Marie Henriette Dior Ndione, Moussa Moïse Diagne, Giulia Mencattelli, Amadou Diallo, El Hadji Ndiaye, Marco Di Domenico, Diawo Diallo, Mouhamed Kane, Valentina Curini, Ndeye Marieme Top, Maurilia Marcacci, Safiétou Sankhe, Massimo Ancora, Barbara Secondini, Valeria Di Lollo, Liana Teodori, Alessandra Leone, Ilaria Puglia, Alioune Gaye, Amadou Alpha Sall, Cheikh Loucoubar, Roberto Rosà, Mawlouth Diallo, Federica Monaco, Ousmane Faye, Cesare Cammà, Annapaola Rizzoli, Giovanni Savini, Oumar Faye

**Affiliations:** 1 Virology Department, Institut Pasteur de Dakar, Dakar, BP220 Senegal; 2 Istituto Zooprofilattico Sperimentale dell’Abruzzo e del Molise, Teramo, 64100 Italy; 3 Centre Agriculture Food Environment, University of Trento, San Michele all’Adige, 38010 Italy; 4 Research and Innovation Centre, Fondazione Edmund Mach, San Michele all’Adige, 38010 Italy; 5 Epidemiology, Clinical Research and Data Science Department, Institut Pasteur de Dakar, Dakar, BP220 Senegal; 6 Medical Zoology Department, Institut Pasteur de Dakar, Dakar, BP220 Senegal

**Keywords:** Usutu virus, Next-generation sequencing, Amplicon-based sequencing

## Abstract

Usutu virus (USUV), an arbovirus from the *Flaviviridae* family, genus *Flavivirus*, has recently gained increasing attention because of its potential for emergence. After his discovery in South Africa, USUV spread to other African countries, then emerged in Europe where it was responsible for epizootics. The virus has recently been found in Asia. USUV infection in humans is considered to be most often asymptomatic or to cause mild clinical signs. However, a few cases of neurological complications such as encephalitis or meningo-encephalitis have been reported in both immunocompromised and immunocompetent patients. USUV natural life cycle involves *Culex* mosquitoes as its main vector, and multiple bird species as natural viral reservoirs or amplifying hosts, humans and horses can be incidental hosts. Phylogenetic studies carried out showed eight lineages, showing an increasing genetic diversity for USUV. This work describes the development and validation of a novel whole-genome amplicon-based sequencing approach to Usutu virus. This study was carried out on different strains from Senegal and Italy. The new approach showed good coverage using samples derived from several vertebrate hosts and may be valuable for Usutu virus genomic surveillance to better understand the dynamics of evolution and transmission of the virus.

**Supplementary Information:**

The online version contains supplementary material available at 10.1186/s12985-024-02426-7.

## Introduction

An increasing number of Infectious diseases (re-)emerge annually due to a combination of ecological and socio-economical drivers such as increase in human population density, ageing, travel, urbanization, biodiversity loss and climate change, promoting the evolution and spread of new pathogens [1]. Among them, arboviruses have a significantly impact on human and animal health through their emergence and re-emergence in these populations, representing a significant threat for new epidemics worldwide. [2, 3].

Usutu virus (USUV), an arbovirus from the *Flaviviridae* family, genus *Flavivirus*, has recently gained significant scientific attention, particularly since its emergence in Europe [3]. It was first isolated in 1959 from a *Culex neavei* mosquito caught near the Usutu river in Swaziland, South Africa [4]. The virus belongs to the antigenic serocomplex of Japanese encephalitis, together with Japanese encephalitis virus (JEV), West Nile virus (WNV), and Murray Valley encephalitis virus (MVEV). It is a (+)-strand RNA genome of 11,064 nucleotides encoding a single polyprotein of 3,434 amino acids that is subsequently cleaved into structural (C, prM and E) and non-structural (NS1, NS2A, NS2B, NS3, NS4A, NS4B and NS5) proteins [5].

Similar to other flaviviruses, the natural life cycle of USUV involves *Culex* mosquitoes as its main vector, and multiple bird species as natural viral reservoirs or amplifying hosts [3]. Humans and horses can be incidental dead-end hosts. USUV isolation, detection or serological evidence were reported in various animal species including rodents, shrews, bats, dogs, squirrels, wild boars, roedeers, and lizards, expending the incidental host range [6–27].

The genetic variability of USUV has been explored through phylogenetic studies performed on full-length genomes, as well as on the envelope and NS5 genes [17, 28–31]. These analyses revealed eight distinct USUV lineages designated on the basis of their geographic origin of isolation: Africa 1, 2 and 3; Europe 1, 2, 3, 4 and 5.

After its discovery in South Africa, USUV spread to other African countries including Senegal, Uganda, Central African Republic, Nigeria, Burkina Faso, and Côte d’Ivoire, causing mild sporadic infections in the continent [32]. Outside Africa, it was observed in Europe for the first time in Vienna in 2001 [9], where it was responsible for massive outbreaks in blackbirds (*Turdus merula)* and gray owls *(Strix nebulosa)*. A retrospective study analysing dead birds conducted in the Tuscany region of Italy showed that USUV has been circulating in Europe since 1996 [10]. Between 2014 and 2015, the virus was first isolated in Israel from mosquito pools, marking the first isolation in the Asian continent [33]. Recently, USUV has also caused neurological cases in immunocompetent patients in Croatia, Italy, Germany, Austria and France [31, 34–37]. Indeed, there is increasing evidence that the virus is pathogenic for humans, and might become a growing potential public health issue.

USUV is found in a broad spectrum of animals and mosquitoes, sometimes in co-infection with insect specific viruses or other arboviruses sharing the same reservoirs or vectors [38, IPD unpublished data]. This poses a diagnostic challenge for both serology and molecular biology tests. We therefore set up an amplicon-based system for the specific sequencing of USUV. The sequencing approach, used extensively during the COVID-19 pandemic, allowed health authorities and the scientific community to quickly monitor SARS-CoV-2 introductions and identify new variants for appropriate countermeasures and better management of the pandemic [39]. This tool will enable the acquisition of complete genomes of USUV strains circulating at known and unknown vectors, reservoirs, or hosts, allowing for better genetic diversity assessment and genomic surveillance in Africa, Europe and other parts of the world where the virus can emerge.

## Methods

### Primers design for USUV tiled amplicons-based sequencing systems

Two non-overlapping pools of USUV targeting primers were designed in IPD to perform multiplexed PCR reactions, using the web-based tool entitled Primal Scheme [40] and the USUV reference genome (accession number: MT188658.1) as template. Approximately, 400 bp tiled amplicons were generated along the targeted genome. An alignment of USUV sequences available on Genbank were then used to identify nucleotide mismatches for potential correction on ambiguous sites of each primer to ensure both good coverage and high specificity to USUV lineages.

### USUV primer pools validation

#### Sequencing of USUV isolates

The designed primer systems were challenged for amplicon-based whole genome sequencing of well characterized USUV isolates from Senegal and Italy. The sequencing experiments were undertaken both by the teams in Senegal and Italy with their local isolates. USUV strains from Senegal were obtained by infecting C6/36 cell monolayers with homogenized mosquito pools, as previously described [41]. Isolates from Italy were obtained from birds’ internal organs and mosquito homogenates after two to three passages on Vero monolayer cell lines followed by an infection on C6/36 cell lines. A cut-off of 95% horizontal coverage was chosen as a good metric for inclusivity.

#### Specificity and sensitivity of the USUV amplicon based-sequencing systems

The specificity of USUV amplicon was assessed by performing the experiment on several other arboviruses: Rift Valley Fever virus (RVFV), Yellow Fever virus (YFV), Zika virus (ZIKV), Dengue 2 virus (DENV-2), Wesselsbron virus (WSLV), Kedougou virus (KDGV), WNV and Chikungunya virus (CHIKV) as previously described [42]. Moreover, the sensitivity of the approach was evaluated using serial dilutions of USUV isolates at different concentrations (10^6–10^2 RNA copies/µl). Each concentration was sequenced in triplicate.

#### Validation on USUV field samples

Sequencing efforts were conducted on USUV positive samples from mosquitoes and birds collected in both Italy and Senegal. CT values of the different samples were determined by RT-qPCR using a consensus USUV assay in Senegal [43] and a molecular USUV assay [44] in Italy before sequencing. Briefly, in Senegal viral RNA extraction was performed with the QIAamp viral RNA mini kit (Qiagen, Heiden, Germany) according to the manufacturer’s instructions. Viral RNAs were amplified by qRT-PCR using the Quantitect Reverse Transcription Kit (Qiagen, Heiden, Germany) according to the manufacturer’s instructions and a specific real-time RT-PCR assay to Identify Usutu Virus [43]. In Italy, Viral RNA was extracted by using the MagMAX CORE Nucleic Acid Purification KIT (Applied Biosystem, Thermo Fisher Scientific, Life Technologies Corporation, TX, USA) and amplified by a rapid and specific real-time RT-PCR assay to Identify Usutu Virus [44], by using the Superscript III Platinum OneStep qRT-PCR System (Invitrogen) according to the manufacturer’s instructions.

#### Next generation sequencing and genome assembly

Viral RNAs were extracted using the QIAamp viral RNA mini-kit (QIAGEN, Hilden, Germany) and were reverse-transcribed into cDNAs using the Superscript IV Reverse Transcriptase enzyme (ThermoFisher Scientific, Waltham, MA, USA). The synthesized cDNAs served as templates for direct amplification to generate approximately 400 bp amplicons tiled along the genome using two non-overlapping pools of USUV targeting primers at 10 nM and Q5^®^ High-Fidelity 2X Master Mix (NEB, NEW ENGLAND, Biolabs).

In Senegal, libraries were then synthesized by tagmentation using the Illumina DNA Prep kit and the IDT^®^ for Illumina PCR Unique Dual Indexes. After a cleaning step with the Agencourt AMPure XP beads (Beckman Coulter), libraries were quantified using a Qubit 3.0 fluorometer (Invitrogen Inc., Waltham, MA) for manual normalization before pooling in the sequencer. Clusters generation and sequencing were performed on an Illumina MiSeq instrument with 2 × 300-nt read-length. Consensus genomes were generated using the nextflow (v21.10.6) based nf-core viral reconstruction (v2.5) pipeline (https://github.com/nf-core/viralrecon) from the standardized nf-core pipelines [44, 45].

In Italy, amplified DNA was diluted to obtain a concentration of 100–500 ng, then used for library preparation with an Illumina DNA prep kit, and sequenced with a NextSeq 500 (Illumina Inc., San Diego, CA, USA) using a NextSeq 500/550 Mid Output Reagent Cartridge v2, 300 cycles, and standard 150 bp paired-end reads. After quality control and trimming with Trimmomatic v0.36 (Usadellab, Düsseldorf, Germany) (Bolger et al., 2014) and FastQC tool v0.11.5 (Bioinformatics Group, Babraham Institute, Cambridge, UK) [47, 48] reads were *de novo* assembled using SPADES v3.11.1 (Algorithmic Biology Lab, St Petersburg, Russia) [49]. The contigs obtained were analyzed with BLASTn to identify the best match reference. Mapping of the trimmed reads was then performed using the iVar computational tool [50] to obtain a consensus sequence.

#### USUV phylogenetic tree

A phylogenetic tree The newly generated USUV genomes were analysed with other whole genomes available in Genbank. A phylogenetic tree was generated with the strains obtained during this study (horizontal coverage greater than or equal to 95%) with complete genomes available on Genbank. Indeed, complete genomes of strains of each lineage described for USUV (Africa 1, 2, 3 et Europe 1, 2, 3, 4, 5) [30–32] were chosen according to availability. Sequences were aligned using MAFFT [51], and the alignment was run under the best model in IQ-TREE [52]. The maximum-likelihood (ML) phylogenetic tree was visualized using Figtree V1.4.4 [53].

## Results

### USUV oligonucleotide primer pools

Overall, thirty-five overlapping oligonucleotide primer pairs covering almost the whole genome of USUV were obtained after the design based on a USUV reference genome (accession number: MT188658.1).

The primers set was subsequently compared to an alignment of twenty-one sequences representing the different USUV lineages (supplementary material S1). In order to cover a maximum of lineages while maintaining a balance for specificity, degeneration was then added in relevant ambiguous sites on each primer. The list of USUV primers can be found in Table 1.

**Table 1:** Sequences of the USUV primers set

USUV_1_LEFT	CGTGAGCTCTACTACTTCATATTGGT
USUV_1_RIGHT	CTAGTCCTGGTCCATTGTTGCC
USUV_2_LEFT	ACCTGACTAGCTTTAAAAAGGAATTAGG
USUV_2_RIGHT	TCGAATGTCTGGTTCTTGTGCA
USUV_3_LEFT	GGATGCAGGAAATGACCCAGAA
USUV_3_RIGHT	ACCTTCCAGAACCACGTCRA
USUV_4_LEFT	TGCTTACAGCTTCAACTGCCTT
USUV_4_RIGHT	TTTTCCGGTTGGATCATTCGGC
USUV_5_LEFT	ATGGCTGTGGACTATTTGGCAA
USUV_5_RIGHT	AGTRTCTCTCTATTTCTCCAATTTGAGCT
USUV_6_LEFT	GCACCAAACATTTCCTTGTCCA
USUV_6_RIGHT	TGWTGCCACGATGGAAATTGGA
USUV_7_LEFT	CGGCATGTGTACGGAAAAGTTT
USUV_7_RIGHT	ATTGAAAATCCCTCCGACCGAC
USUV_8_LEFT	TGGAAAAGCGTTCATCACCACT
USUV_8_RIGHT	CCAGYTGTTTTGGTGTCTCAGG
USUV_9_LEFT	GCTGTGGACAAGGGATCTTCAT
USUV_9_RIGHT	CCTTTTTACGTCGGGGCATTCT
USUV_10_LEFT	CTGGGGAAAGAGCTTGGTGTTC
USUV_10_RIGHT	GATTGCTTTTTGGTCCAGCGAG
USUV_11_LEFT	GCCTGAGACACACACTCTTTGG
USUV_11_RIGHT	ATGTCACTCCGGTAGGCACTAA
USUV_12_LEFT	TGGATGTTGGTATGGAATGGARAT
USUV_12_RIGHT	AGCAGGATGTTCTCTTGGTTYG
USUV_13_LEFT	GCCGCCTTTAAAATCCAACCAG
USUV_13_RIGHT	GGARAACTGCCCTGTTGATGTT
USUV_14_LEFT	CATYTGCAGCCTGATAGGGGAG
USUV_14_RIGHT	GGGTCGTTGATAAGGTGGAAGT
USUV_15_LEFT	GATGCAGCCATAACTGGAACCA
USUV_15_RIGHT	GTGAGCCTTCCTTCACCACTTC
USUV_16_LEFT	GTGGGCGTCATGTATGAAGGAG
USUV_16_RIGHT	TTCTTCTCTTTCCCCTTGCACG
USUV_17_LEFT	ATCGTGGGCTTGTACGGGAAT
USUV_17_RIGHT	TTAGAGGTGACATGAGTCTGTGG
USUV_18_LEFT	CAGCGGTCAACAGAGAGCAYAG
USUV_18_RIGHT	TTCCCGCTCTTTGAAGACACTG
USUV_19_LEFT	ATACACCGGGAAAACAGTCTGG
USUV_19_RIGHT	CGATCGTGTCATCTTCGCTTGT
USUV_20_LEFT	TGCCCAGAGGAGAGGAAGAGTA
USUV_20_RIGHT	GCCYGTGCGGGTGACAATYT
USUV_21_LEFT	CCGACAGGAAGTGGTGTTTTGA
USUV_21_RIGHT	TCTGAACGAGGAGCAAGAAAACT
USUV_22_LEFT	CATGGCCTTTGAAGAGTTGCC
USUV_22_RIGHT	AGAGCACTCTGTGGTTGACGT
USUV_23_LEFT	GTGGTGGCTGCAAATGAATACG
USUV_23_RIGHT	GYAGCATGTAGCCATAGTGRAG
USUV_24_LEFT	CTTGGTCTTTTTGGGATGYTGG
USUV_24_RIGHT	GATGACGTGACAAAGTCCGGTC
USUV_25_LEFT	CATCCTCATATCAGCGGCACTC
USUV_25_RIGHT	CCACCTTGCCAATTGGTTTGAC
USUV_26_LEFT	GGRAACAAAACTGGAGGACACC
USUV_26_RIGHT	TGCAGAACTCTCTTGGTCCTCT
USUV_27_LEFT	AGTGCTGAGGTGGAAGAACAAC
USUV_27_RIGHT	TCCTGGCTTTGATCTTCTCYTG
USUV_28_LEFT	ACGAGGAGGATGTTAACCTTGG
USUV_28_RIGHT	GAGAAAAGCCCACAGCCAATTG
USUV_29_LEFT	AGAAAAGGTTGACACCAAGGCC
USUV_29_RIGHT	CARGGCTTCAAACTCCAGRAAT
USUV_30_LEFT	GAGAGTTTGGCAAAGCGAAAGG
USUV_30_RIGHT	CATCCATGACGGTCTTCCCATC
USUV_31_LEFT	ACAACTRGCCAGAGCAATCATT
USUV_31_RIGHT	ACTCTGGCACGTCTTTTCTGAC
USUV_32_LEFT	GTTGTCAAGCCCCTGGATGATC
USUV_32_RIGHT	ATGCACTGACCATGACGTCCT
USUV_33_LEFT	AGATCTGAGGCTGATGGCAAAC
USUV_33_RIGHT	TTACAAAACCCTGTCCTCTTGGA
USUV_34_LEFT	ACCAGGTGAGGGCAATTATTGG
USUV_34_RIGHT	GACGCTTCCAATAAGCAGGGTC
USUV_35_LEFT	CGGACTGGGTTAACAAAGCTGG
USUV_35_RIGHT	TGCCTTGTGGTTGATGTTGGAA

The Table 1 shows the sequences of the USUV primers set designed in this study

### USUV primers set validation

#### Inclusivity test

Overall, three strains from Italy and ten for Senegal, with Ct values varying from 10 to 33, were used to perform the USUV primer’s inclusivity test. The tiled-amplicon whole genome sequencing, undertaken on both strains from Senegal and Italy, obtained 95 − 100% horizontal coverage with genome length between 10,494nt and 10,837nt (Table 2).

**Table 2:** Inclusivity test of the USUV primers

RT-PCR Ct value			# Total trimmate reads	# USUV reads	% HCoverage	VCoverage	Consensus sequence length
*Viral strain USUV Italy*	10		1.997.126	56.4902	100	6053.7	10.837
	20		1.909.064	482.135	100	5360.97	10.835
	16		2.056.663	553.137	100	6041.88	10.837
	**N of replicates with Coverage ≥ 95%**				**3/3 (100 )**		
*Viral strain USUV Senegal*	33		694.838	225.462	95	3041.56	10.494
	28		854.870	304.085	97	4033.3	10.494
	27		556.582	223.863	97	3024.04	10.499
	31		523.901	197.085	95	2715.43	10.494
	29		769.757	247.559	95	3073.1	10.499
	32		3.250.194	605.236	97	5460.49	10.498
	13		628.539	281.283	99	3729.34	10.695
	13		679.204	289.399	100	3803.38	10.825
	14		379.968	188.337	100	2479.05	10.836
	14		445.022	221.901	99	2975.98	10.699
	**N of replicates with Coverage ≥ 95%**				**10/10 (100)**		

The Table 2 shows the results obtained after sequencing of three and ten USUV strains from Italy and Senegal respectively, for inclusivity test of the USUV primers. Ct values of tested strains, number of total reads and specific USUV reads, horizontal and vertical coverages and consensus sequence length obtained after sequencing for each strain were presented in this table

VCoverage = vertical coverage

HCoverage = horizontal coverage

#### Specificity test

In order to assess the specificity of the USUV targeted approach, six *Flaviviruses* (YFV, ZIKV, DENV-2, WSLV, KDGV, WNV), one *Phlebovirus* (RVFV), and one *Alphavirus* (CHIKV) were tested with the USUV primer’s set for amplicon-based whole genome sequencing.

All the samples failed bowtie2 1000 mapped read threshold and no consensus genome could be assembled.

#### Sensitivity test

One representative USUV isolate (accession number: ON032476) was chosen to assess the primers set limit of detection under optimal conditions. Serial dilutions from 10^6 to10^2 cp/µl were processed in triplicate for sequencing. The system was able to capture more than 95% of USUV whole genome until 10^4 cp/µl. At 10^3 cp/µl, horizontal coverage was between 92 and 96% while 83–88% of the USUV sequence was completed at 10^2 cp/µl (Table 3).

**Table 3:** Sensitivity test of the USUV primers

Quantity value (cp/uL)	# Total trimmate reads	# WNV reads	% HCoverage	VCoverage	Consensus sequence length
10^6	1.189.449	376.979	100	4607.17	10.837
10^6	1.269.455	403.033	100	4838.74	10.836
10^6	1.198.499	392.297	100	4745.8	10.837
**N of replicates with Coverage ≥ 95%**			**3/3 (100)**		
10^5	1.240.823	379.133	99	4581.63	10.837
10^5	1.073.364	351.179	99	4289.55	10.807
10^5	1.235.377	386.080	99	4624.55	10.837
**N of replicates with Coverage ≥ 95%**			**3/3 (100)**		
10^4	1.392.113	381.234	99	4339.31	10.838
10^4	1.169.792	349.199	97	4178.8	10.499
10^4	1.094.369	334.163	96	4135.57	10.499
**N of replicates with Coverage ≥ 95%**			**3/3 (100)**		
10^3	641.826	223.790	96	2848.31	10.499
10^3	695.820	238.992	95	3069.17	10.351
10^3	462.528	182.763	92	2441.9	10.502
**N of replicates with Coverage ≥ 95%**			**2/3 (66%)**		
10^2	85.909	50.388	88	709.483	10.348
10^2	44.720	28.768	88	406.636	10.346
10^2	37.560	24.606	83	366.618	10.340
**N of replicates with Coverage ≥ 95%**			**0/3 (0%)**		

The Table 3 shows the evaluation of sensitivity of USUV primers set. The number of total reads and specific USUV reads, horizontal and vertical coverages and consensus sequence length obtained after sequencing of serial dilutions of an USUV strain were presented in this table

VCoverage = vertical coverage

HCoverage = horizontal coverage

#### USUV Set primers validation on field samples

Forty-three USUV homogenates with known RT-qPCR Ct values ranging from 15 to 35 were selected for targeted-sequencing. The homogenates were obtained from mosquito pools and bird internal organs. The horizontal coverage of the samples ranged from 58 to 100%, corresponding to Ct values between 15 and 35, and 62% of the samples reached coverage between 95 and 100% with Ct values ranging from 15 to 27. Two samples with Ct values of 27 and 30 failed the sequencing process, indicating that factors other than the viral load could be involved in the consensus genome assembly. Interestingly, the amplicon-based sequencing approach was also performed on six USUV positive samples from Italy that were co-infected with WNV-Lineages 1 or 2, giving an USUV H-coverage of 92–100%, despite having a lower viral load compared to WNV. Similarly, relative good H-coverage (74–93%) was obtained on six mosquito samples from Senegal with reported viral co-infections either by flaviviruses, alphaviruses, or mesoniviruses. All these results are summarized in Tables 4 and 5.

**Table 4:** Test of the USUV primers with USUV homogenates

Viral homogenate USUV – sample number	Host	RT-PCR Ct value	# Total trimmate reads	# USUV reads	% HCoverage	VCoverage	Consensus sequence length
1	*Turdus merula*	15	2.465.611	565.498	100%	6657.9	11.008
2	*Turdus merula*	15	1.424.014	1.373.423	99%	6748.44	11.033
3	*Culex pipiens*	16	3.867.757	543.717	99%	5625.6	10.773
4	*Culex pipiens*	17	4.303.602	600.693	100%	5499.75	10.835
5	*Culex pipiens*	18	2.991.770	564.111	99%	5770.54	10.826
6	*Larus michaellis*	18	4.033.463	753.511	100%	6643.55	11.003
7	*Culex pipiens*	19	2.388.992	532.830	97%	6048.34	11.013
8	*Sturnus vulgaris*	21	3.897.031	557.169	100%	5491.77	10.837
9	*Culex pipiens*	21	2.639.206	431.426	100%	4726.96	10.837
10	*Turdus merula*	22	2.176.309	483.373	100%	5880.38	11.002
11	*Culex pipiens*	22	5.122.948	527.184	100	4654.43	10,836
12	*Culex pipiens*	23	3.169.173	414.895	97%	4760.21	10.497
13	*Culex pipiens*	23	4.272.994	650.865	100%	6095.87	10.837
14	*Culex pipiens*	23	2.366.094	544.141	97%	6171.29	11.020
15	*Culex pipiens*	23	2.947.599	458.013	100%	4779.29	10.837
16	*Culex pipiens*	23	14.286.460	781.029	95%	5859.88	10.649
17	*Culex pipiens*	23	3.402	2.069	78%	355.225	10.484
18	*Passer domesticus*	24	845.671	246.117	89%	4373.47	10.999
19	*Columba palumbus*	24	2.209.345	493.383	98%	5956.48	11.002
20	*Culex pipiens*	25	2.568.496	546.673	97%	6063.81	11.004
21	*Culex pipiens*	25	3.263.678	428.103	97%	4658.68	10.498
22	*Culex pipiens*	25	11.784.307	816.887	97%	6032.09	10.505
23	*Culex pipiens*	25	3.619.530	650.199	97%	6145.91	10.837
24	*Turdus merula*	26	1.691.299	372,666	93%	5409.48	11.002
25	*Culex pipiens*	26	4.181.786	519.835	100%	5420.14	10.837
26	*Culex pipiens*	26	1.516.361	250.029	94%	3403.15	10.500
27	*Culex pipiens*	27	3.383.309	308.081	81%	3944.35	9.867
28	*Culex pipiens*	27	1.516.324	484.629	92%	5089.83	10.501
29	*Columba palumbus*	27	1.418.856	263.207	93%	4676.13	10.992
30	*Phasianus colchicus*	27	177.310	0	0	0	0
31	*Culex pipiens*	27	2.613.0567	592.806	97%	4734.12	10.515
32	*Culex pipiens*	28	2.108.171	197.121	93%	2403.54	10.495
33	*Otus scops*	30	210.181	95.737	86%	2532.56	10.678
34	*Culex pipiens*	30	348.624	0	0	0	0
35	*Columba palumbus*	31	2.358.055	163.177	92%	3980.69	10.679
36	*Otus scops*	33	101.403	16.512	84%	348.585	10.642

The Table 4 shows the results obtained after sequencing of USUV homogenates (mosquitoes and birds) with the USUV primers set. The hosts species, Ct values of tested strains, number of total reads and specific USUV reads, horizontal and vertical coverages and consensus sequence length obtained after sequencing were presented in this table

VCoverage = vertical coverage

HCoverage = horizontal coverage

**Table 5:** Quantitative and conventional RT-PCR results of samples with multiple viral species

Viral homogenate USUV – sample number	Host	USUV RT-PCR Ct-value	WNV L1 RT-PCR Ct-value	WNV L2 RT-PCR Ct-value	MESOV RT-PCR Ct-value	BAGV RT-PCR Ct-value	BARKV RT-PCR Ct-value	SINDV Conventional RT-PCR	ONNV Conventional RT-PCR
13	*Culex pipiens*	23	-	21	-	-	-	-	-
15	*Culex pipiens*	23	38	-	-	-	-	-	-
25	*Culex pipiens*	26	-	26	-	-	-	-	-
26	*Culex pipiens*	26	-	29	-	-	-	-	-
28	*Culex pipiens*	27	-	27	-	-	-	-	-
30	*Phasianus colchicus*	27	-	32	-	-	-	-	-
31	*Culex pipiens*	27	-	24	-	-	-	-	-
38	*Culex neavei*	30	-	-	15	-	-	+	-
39	*Culex neavei*	28	-	-	-	32	27	-	+
40	*Culex neavei*	27	-	-	-	-	24	-	+
41	*Culex neavei*	34	-	-	-	-	28	-	+
42	*Culex neavei*	33	26	-	-	-	29	-	+
43	*Culex neavei*	24	25	-	-	-	26	-	+

The Table 5 shows the quantitative and conventional RT-PCR results of samples with multiple viral species including USUV. Host species, Ct values and/or results of conventional RT-PCR of different viruses detected in each strain were presented in this table

(-): Negative

(+): Detected by conventional RT-PCR

SINDV: Sindbis virus; MESOV: Mesonivirus virus; BAGV: Bagaza virus; WNV: West nile virus; BARKV: Barkedji virus; ONNV: O’nyong’nyong virus

#### Usutu virus phylogenetic tree

**Fig. 1:**
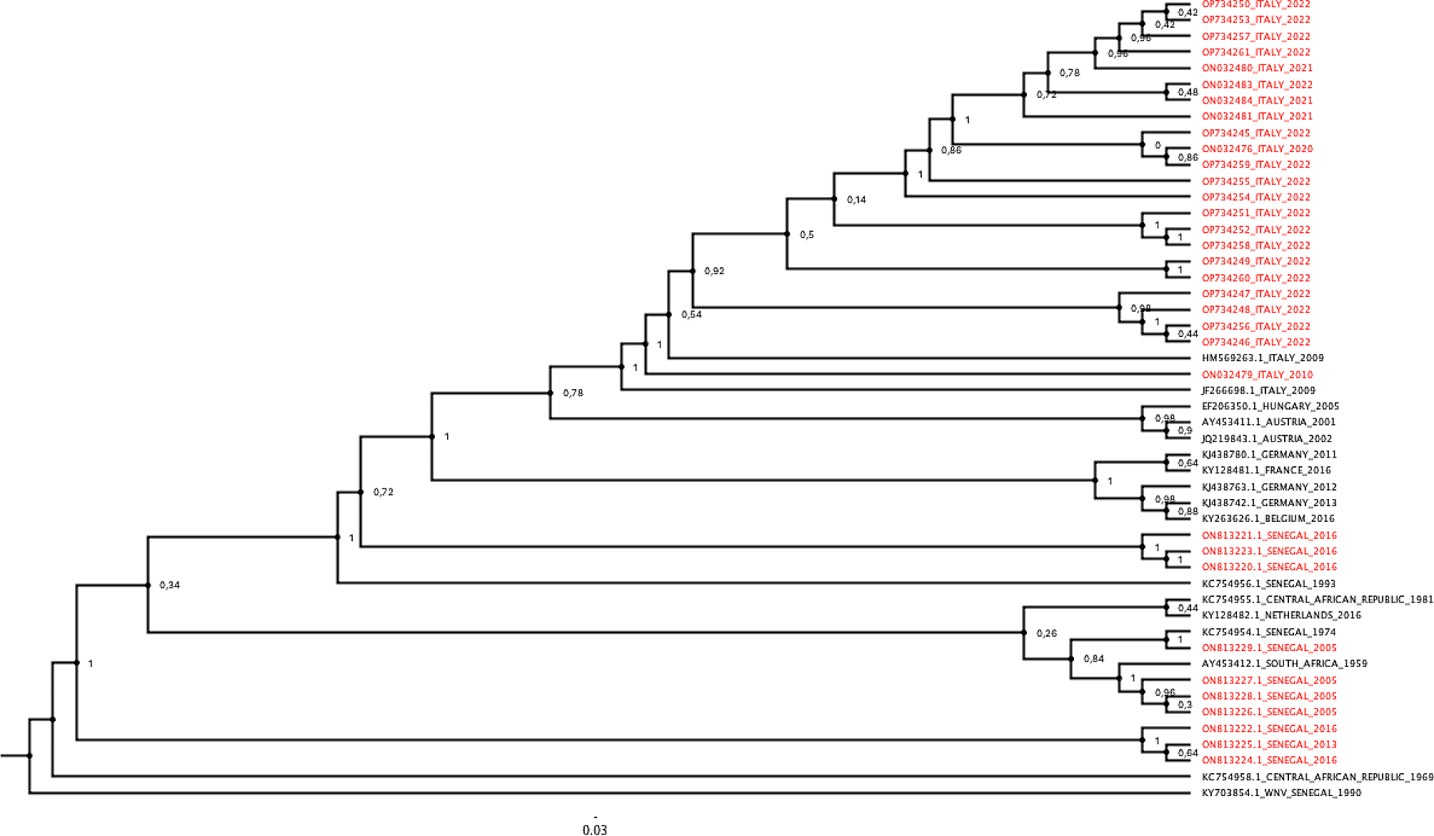
USUV phylogenetic tree. Newly obtained USUV (in red) strains and available complete genomes (in black) of USUV in Genbank were analysed to obtain a phylogenetic tree to compare sequences each others

The phylogenetic tree obtained after analysis of newly USUV strains and USUV available complete genomes in Genbank, confirms that the complete genomes sequences obtained belong to USUV and that these strains have a resemblance to the old strains (Fig. 1).

## Discussion

Arboviruses constitute a broad group of viruses with a strong impact on human and animal health. In order to prevent the underreporting and underestimation of these viruses, there is a strong need of a reliable integrated surveillance system at the animal, human and vector interface. High-throughput sequencing approaches, as the nucleic acid metagenomics; the hybrid capture using specific biotinylated probes; the multiplex PCR-based target enrichment; or the amplicon-based protocol might be crucial to improve viral diagnosis and better understand the genetic diversity of viruses, enabling the implementation of appropriate countermeasures [42]. Specifically, the amplicon-based sequencing approach, allowing targeted-sequencing, has been extensively used in the recent past for SARS-CoV-2 and WNV genomic surveillance to monitor the virus introductions and local transmission, aiding in understanding the global diffusion network and viral evolution [54]. This method offers a solution to sequencing constraints such as the host genomic background and low viral loads [55].

USUV, an arbovirus causing neurological disease in human, has become widespread in Africa and Europe [32, 56] and has emerged in Asia [33]. Several detections of the virus in humans, mosquitoes and animals have been recorded in recent years across these three continents. USUV genetic lineages are geographically specific, enabling genomic surveillance to track viral strain diffusion patterns. In this study, we propose a whole genome amplicon-based sequencing approach for Illumina technology for USUV, to establish an efficient genomic surveillance system for monitoring the emergence and re-emergence of this arbovirus that, widespread in Africa and Europe, is causing neurological disease in humans and which evolution and expansion might represent a potential public health future threat worldwide [32, 56]. The method was validated in vertebrates and mosquitoes from Senegal and Italy using specific primers designed based on different USUV lineages. Nearly complete genomes sequences of USUV strains from both countries were obtained with good primer sensitivity and specificity. In addition, USUV samples co-infected with other viruses were sequenced with good horizontal and vertical coverages. In view of these results, this sequencing method can be used for USUV monitoring, directly from mosquito and bird homogenates. However, additional comparative studies could be carried out to assess the described USUV primers set and refine the observed results.

In the literature, many phylogenetic studies which have shown that USUV can have several lineages including Africa 1 to 3 and Europe 1 to 5 have been essentially based on the NS5 gene of the virus [28–31, 57]. New phylogenetic studies based on complete genomes of all detected strains in African and European countries using this tool could allow to better determine the diversity of this virus.

Overall, this novel amplicon based sequencing tool can support USUV genomic surveillance worldwide.

### Electronic supplementary material

Below is the link to the electronic supplementary material.

Supplementary Material 1

## Data Availability

The data presented in this study are available in the results section of the article as well as in the supplementary materials. No new data were created or analyzed in this study.

## Abbreviations

USUV: Usutu Virus
JEV: Japanese Encephalitis Virus
WNV: West Nile Virus
MVEV: Murray Valley Encephalitis Virus
RVFV: Rift Valley Fever Virus
YFV: Yellow Fever Virus
ZIKV: Zika Virus
DENV-2: Dengue Virus Lineage 2
WSLV: Wesselbron Virus
KGGV: Kedougou Virus
CHIKV: Chikungunya Virus
MESOV: Mesonivirus Virus
BAGV: Bagaza Virus
BARKV: Barkedji Virus
SINDV: Sindbis Virus
ONNV: O’Nyong’Nyong
